# Investigation of the Therapeutic Potential of Organic Nitrates in Mortality Reduction Following Acute Myocardial Infarction in Hyperlipidemia Patients: A Population-Based Cohort Study

**DOI:** 10.3390/jpm14010124

**Published:** 2024-01-22

**Authors:** An-Sheng Lee, Chung-Lieh Hung, Thung-Shen Lai, Ching-Hu Chung

**Affiliations:** 1 Department of Medicine, Mackay Medical College, New Taipei City 25245, Taiwan; anshenglee@mmc.edu.tw; 2 Division of Cardiology, Departments of Internal Medicine, MacKay Memorial Hospital, Taipei 10449, Taiwan; jotaro3791@mmc.edu.tw; 3 Institute of Biomedical Sciences, Mackay Medical College, New Taipei City 25245, Taiwan; lai00002@mmc.edu.tw

**Keywords:** hyperlipidemia, acute myocardial infarction, organic nitrates, beta-blockers, all-cause mortality

## Abstract

Background: Dyslipidemia is a known risk factor for cardiac dysfunction, and lipid-lowering therapy with statins reduces symptoms and reduces hospitalization related to left ventricular heart failure. Acute myocardial infarction (AMI) is a cause of morbidity and mortality worldwide. In this study, we aimed to determine the real-world AMI treatment drug combination used in Taiwan by using the NHI database to understand the treatment outcomes of current clinical medications prescribed for hyperlipidemia patients with AMI. Methods: Using the NHI Research Database (NHIRD), we conducted a retrospective cohort study that compared different treatments for AMI in hyperlipidemia patients in the period from 2016 to 2018. We compared the survival outcomes between those treated with and without organic nitrates in this cohort. Results: We determined that most hyperlipidemia patients were aged 61–70 y (29.95–31.46% from 2016 to 2018), and the annual AMI risk in these patients was <1% (0.42–0.68% from 2016 to 2018). The majority of hyperlipidemia patients with AMI were women, and 25.64% were aged 61–70 y. Receiving organic nitrates was associated with lower all-cause mortality rates (HR, 95% CI, *p*-value = 0.714, 0.674–0.756, *p* < 0.0001). After multivariate analysis, the overall survival in four groups (beta-blockers, beta-blocker + diuretics, diuretics, and others) receiving an organic nitrate treatment was significantly higher than in the groups that were not treated with organic nitrates (beta-blockers HR = 0.536, beta-blocker + diuretics HR = 0.620, diuretics HR = 0.715, and others HR = 0.690). Conclusions: The survival benefit was significantly greater in patients treated with organic nitrates than in those treated without organic nitrates, especially when combined with diuretics. A combination of organic nitrates could be a better treatment option for hyperlipidemia patients with AMI.

## 1. Introduction

Hyperlipidemia is a general term for the imbalance of several cholesterol levels, including abnormal blood levels of low-density lipoprotein cholesterol (LDL-C) and high-density lipoprotein cholesterol (HDL-C). This lipoprotein cholesterol imbalance is highly associated with the risk of cardiovascular events. The prevalence of hyperlipidemia has increased in recent years owing to progressive economic development and lifestyle changes in Taiwan [1]. A population-based study has also reported a significant increase in hypercholesterolemia in the general population in Taiwan [2]. The age-adjusted prevalence of hyperlipidemia was 9.7% in the adult population in 2012, according to a national survey in Taiwan. Based on the evaluation of hyperlipidemia prevalence in Taiwan, it has become a key issue for clinicians to understand its epidemiology, the risk of CV disease, and its treatment in Taiwan.

AMI is one of the top causes of death and ill health globally [3,4]. An interaction study reported that the risk of MI is attributable to CV risk factors like hyperlipidemia, hypertension, psychosocial stress, and alcohol withdrawal [5]. Several clinical studies have indicated a positive association between AMI incidents and cholesterol, and they also found that LDL-C-lowering therapy can reduce the risk of AMI incidents and mortality [6,7]. As a result, related guidelines recommend high-intensity statin therapy for LDL-C reduction to reduce the risk of AMI [8]. According to the study by Christensen et al., the overall incidence rate of MI in the whole population decreased in both sexes from 2005 to 2021 (females: 143 to 80; males: 243 to 174 (per 100,000 persons)) [9]. However, the overall incidence rate of AMI in hyperlipidemia patients is still unknown.

There are several medications recommended for long-term therapies of AMI. ACE inhibitors and beta-blockers may provide benefits for acute myocardial infarction, and intravenous glyceryl trinitrate probably also has a role. ACE inhibitors are recommended for patients with LVEF impairment or who experience heart failure in the early phase [10,11]. The mechanism of beta-blockers for AMI involves diminishing the myocardial oxygen demand by decreasing the heart rate, systemic arterial pressure, and myocardial contractility. The effects of nitrates involved in AMI include reducing the preload and afterload, relaxation of the epicardial coronary arteries, and dilation of collateral vessels [12,13]. Long-term nitrate therapy is associated with a modest hemodynamic benefit in patients with a low left ventricular ejection fraction [14]. Although any of the combinations above are recommended for use in AMI treatment, the survival outcomes and sequential benefits of these combinations are not well studied. We conducted this retrospective cohort study to determine the real-world AMI treatment drug combinations prescribed in Taiwan and understand their treatment outcomes in hyperlipidemia patients with AMI.

## 2. Materials and Methods

### 2.1. Data Source and Study Inclusion

**Figure 1:**
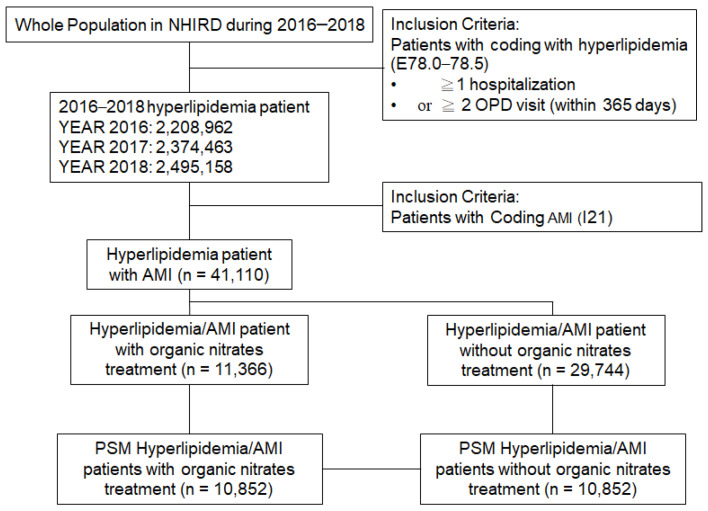
Demographic data of hyperlipidemia patients.

This was a retrospective population-based study conducted by accessing claim records from the National Health Insurance Research Database (NHIRD) from 2016 to 2018. Frequently used for epidemiology and outcomes research [15,16], the NHIRD of Taiwan is a public database available through formal application and approval by the Health and Welfare Data Science Center of the Ministry of Health and Welfare, Taiwan (https://dep.mohw.gov.tw/DOS/np-2500-113.html, accessed on 1 November 2023). Since 2017, the NHI has had contracts with 93.2% of the healthcare facilities in the country, and it is mandatory for doctors at these facilities to report claims data from each patient visit. Thus, the NHIRD database contains the details of all the beneficiaries among most of the healthcare centers in Taiwan [17]. By searching electronic medical records from the NHIRD between 1 January 2016 and 31 December 2018, we retrieved information about patients with a principal diagnosis of hyperlipidemia (two or more ambulatory visits or one hospitalization visit) using ICD-10 code E78.0-78.5. Among these hyperlipidemia patients, those with at least one of the ICD-10: I21 criteria after their hyperlipidemia diagnosis were classified as hyperlipidemia patients with AMI (Figure 1). The protocol of this study was approved by the MacKay Memorial Hospital Institutional Review Board, Taiwan (ROC) (Protocol Number: 19MMHIS083e).

### 2.2. Study Cohorts

All patients meeting the initial inclusion criteria were first categorized into treatment groups with and without organic nitrates (ATC code: C01DA organic nitrates) (Isosorbide 5-mononitrate, nitroglycerin, isosorbide dinitrate) and then further categorized according to the prescription drugs and collectively detailed cohorts of interest: (1) angiotensin-converting enzyme inhibitors/angiotensin receptor blockers (ACEIs/ARB; ATC: C09B/C09D); (2) beta-blockers (ATC: C07); (3) beta-blockers + diuretics (ATC: C03 excluding C03D); (4) diuretics; (5) others (excluding medications 1–4). The nitrates or other drugs were prescribed after AMI for secondary treatment.

### 2.3. Assessment

A diagnosis code used within 1 year before the index date for AMI was identified as indicating an underlying disease. The ECI was used to identify comorbidities in patients with hyperlipidemia. The coding is shown in reference [18]. The log-rank test was used to compare the Kaplan–Meier curves of AMI. The overall survival of the groups was calculated from the first day of the procedure to death or 31 December 2018. We adjusted for potential confounders using logistic regression models and reported the results as adjusted hazard ratios and 95% confidence intervals (CIs). Propensity score matching analysis (PSM) was also conducted. The matching criteria used in this study were matched genders, ages, and ECI scores.

### 2.4. Data Analyses

The number of patients in each group was calculated for the overall study population, each cohort, and by age category within each cohort. The log-rank test was used to compare the Kaplan–Meier curves in each group. The overall survival of the groups was calculated from the first day of MI to death. SAS 9.1 (SAS Institute Inc., Cary, NC, USA) was used for data analyses.

## 3. Results

### 3.1. Baseline Characteristics

The entire NHIRD population from 2016 to 2018 was used to establish the hyperlipidemia patient cohort (Table 1). Our inclusion criteria identified 2,208,962 hyperlipidemia patients in 2016 and 2,495,158 cases in 2018. Considering the whole population in Taiwan, the annual prevalence rate for hyperlipidemia ranged from 9.28% in 2016 to 10.42% in 2018. We also identified the rate of acute myocardial infarction among these hyperlipidemia patients. The cases of acute myocardial infarction ranged from 14,688 to 10,388 between 2016 and 2018, and the incidence rates ranged from 0.68% (665 per 100,000) to 0.42% (416 per 100,000). As shown in Table 2, AMI in hyperlipidemia patients was more highly distributed among males (compared with females). AMI patients diagnosed with hyperlipidemia between the ages of 61 and 80 showed higher prevalence rates between the years 2016 and 2018. The mean age and ECI score of AMI patients with hyperlipidemia treated without organic nitrates were higher than those of patients in the organic nitrates group.

### 3.2. Medication Usage of Hyperlipidemia Patients with AMI and Their Survival with or without Organic Nitrates

**Figure 2:**
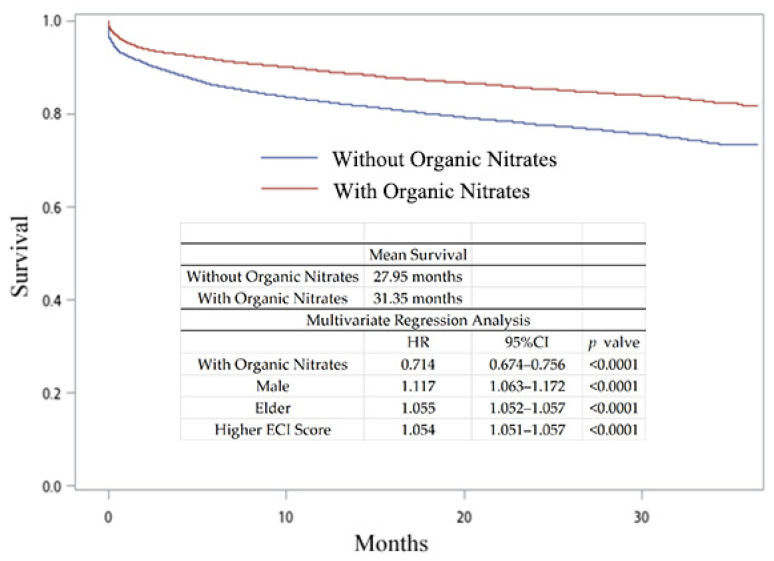
The survival curves and hazard ratios of hyperlipidemia patients with AMI treated with and without organic nitrates.

**Figure 3:**
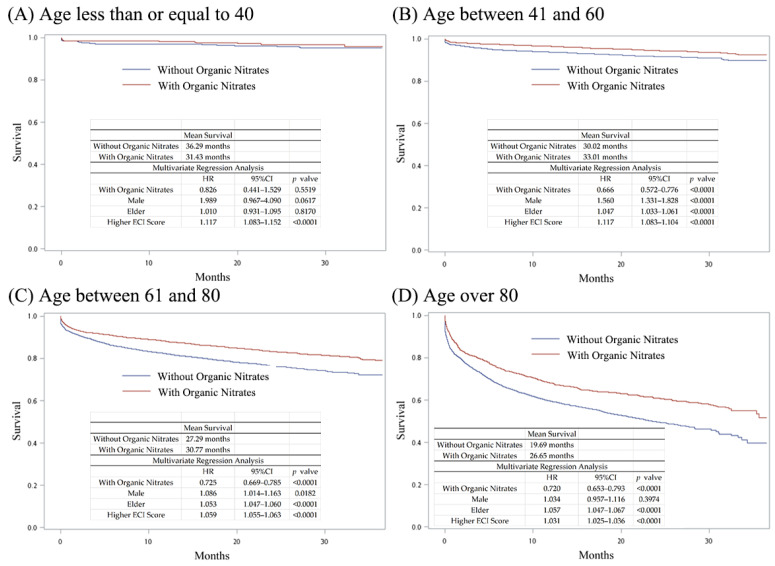
Kaplan–Meier curves and hazard ratios of hyperlipidemia patients with AMI treated with and without organic nitrates in different age groups: (**A**) Age less than or equal to 40 years; (**B**) age between 41 and 60 years; (**C**) age between 61 and 80 years; and (**D**) age over 80.

**Figure 4:**
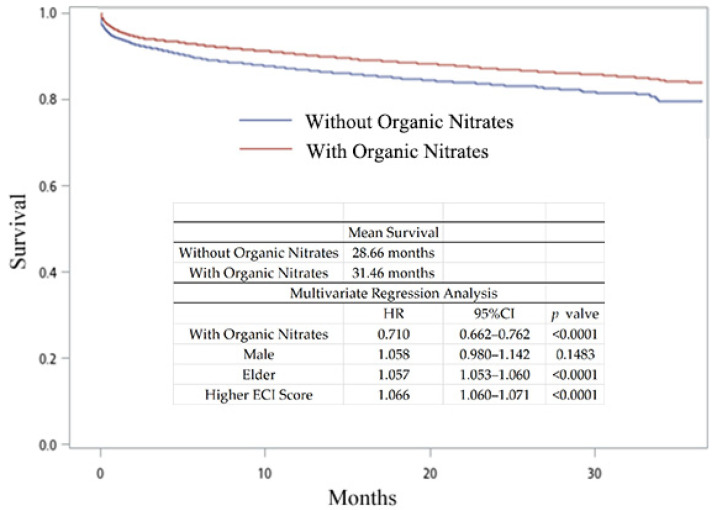
Kaplan–Meier curves and hazard ratios of hyperlipidemia patients with AMI treated with or without organic nitrates after PSM (gender, age, and ECI score).

We hypothesized that NO donor nitrate is essential for hyperlipidemia patients with acute myocardial infarction and is able to provide better survival and sequential outcomes. We divided patients into two groups (AMI treated with or without organic nitrates (ATC code: C01DA organic nitrates)). Only 27.65% (*n* = 11,366) of the hyperlipidemia patients with AMI were treated with organic nitrates (Table 2). We first obtained the mortality rate in hyperlipidemia patients with AMI. As shown in Figure 2, patients receiving organic nitrates exhibited a higher mean survival time (31.35 months, compared with 27.95 months for those not receiving organic nitrates). After multivariate analysis, receiving organic nitrates was associated with lower all-cause mortality rates (HR, 95% CI, *p*-value = 0.714, 0.674–0.756, *p* < 0.0001). Being male and elderly with a higher ECI score was associated with higher all-cause mortality rates. Age may affect survival, and thus we sub-analyzed the patients by age, as shown in Figure 3. According to multivariate analysis, patients receiving organic nitrates exhibited no significant difference in their all-cause mortality rates compared to those not receiving organic nitrates in the age group of ≤40 years (HR, 95% CI, *p*-value = 0.826, 0.441–1.529, *p* = 0.5519). In another age group, receiving organic nitrates was associated with lower all-cause mortality rates, and being male and elderly and having a higher ECI score was associated with higher all-cause mortality rates. To prevent confounding factors from influencing the results, PSM analysis was also conducted. There were 10,852 patients in each group, 77.01% of whom were male, with a mean age of 63.90 years (Table 3). As shown in Figure 4, the patients receiving organic nitrates exhibited a higher mean survival time (31.46 months, compared with 28.66 months for those not receiving organic nitrates). After multivariate analysis, receiving organic nitrates was associated with lower all-cause mortality rates (HR, 95% CI, *p*-value = 0.710, 0.662–0.762, *p* < 0.0001). Older age and a higher ECI score were associated with higher all-cause mortality rates, but there was no significant difference in the all-cause mortality rates among men.

### 3.3. Survival of Hyperlipidemia Patients with AMI Prescribed Different Treatments

**Figure 5:**
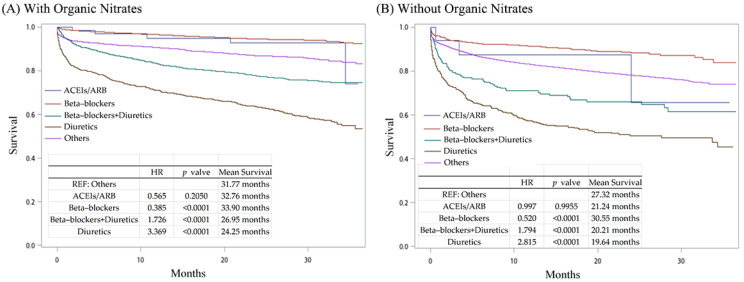
Kaplan–Meier curves for different medications prescribed for hyperlipidemia patients with AMI.

To understand the efficacy of the different treatments, we combined each patient’s survival data to calculate their overall survival. A total of 11,366 patients underwent organic nitrate treatment: ACEIs/ARB (*n* = 72), beta-blockers (*n* = 4312), beta-blockers + diuretics (*n* = 2360), diuretics (*n* = 1263), and others (*n* = 3359). A total of 29,744 patients did not undergo organic nitrate treatment: ACEIs/ARB (*n* = 17), beta-blockers (*n* = 552), beta-blockers + diuretics (*n* = 223), diuretics (*n* = 496), and others (*n* = 28,456). Figure 5 shows the patients’ survival curves. In patients both with and without organic nitrate treatment, only beta-blockers conferred significant survival benefits compared to the other treatment groups (Figure 5A,B). However, the beta-blockers + diuretics and diuretics showed less efficacy, with worse survival profiles compared to the other treatments in both groups. We also found that ACEIs/ARB therapy did not result in a significant difference compared to the other treatments.

### 3.4. The Importance of Organic Nitrates in the Survival of Hyperlipidemia Patients with Acute Myocardial Infarction Prescribed Different Treatments

**Figure 6:**
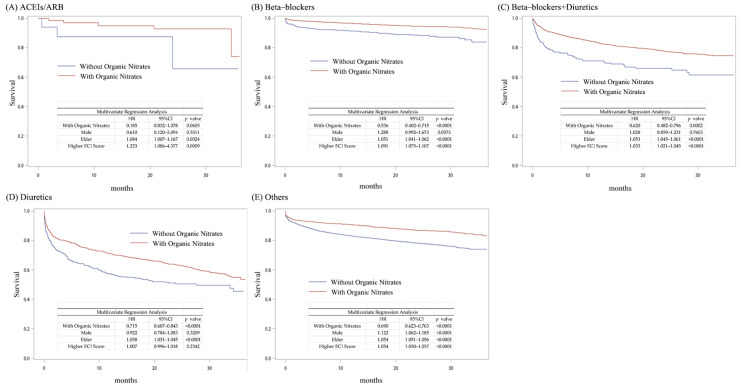
Kaplan–Meier curves of different medications in combination with or without organic nitrates in hyperlipidemia patients with AMI.

We also compared the five medication groups with or without organic nitrate treatment and measured their survival curves to understand the differences among these medications. After multivariate analysis, the overall survival rates in the four groups that received organic nitrate treatment (beta-blockers, beta-blockers + diuretics, diuretics, and others) were significantly higher than in groups without organic nitrate treatment (beta-blockers HR = 0.536, beta-blockers + diuretics HR = 0.620, diuretics HR = 0.715, and others HR = 0.690). Figure 6 shows these patients’ survival curves. Although the survival benefit of combining ACEIs/ARB with organic nitrate treatment was not significant, this group still showed a more favorable trend compared to the group that did not receive organic nitrate treatment. These results indicate that organic nitrates are key medications for hyperlipidemia patients with AMI.

### 3.5. The Difference in NO-Enhancing Agents for the Survival of Hyperlipidemia Patients with Acute Myocardial Infarction

**Figure 7:**
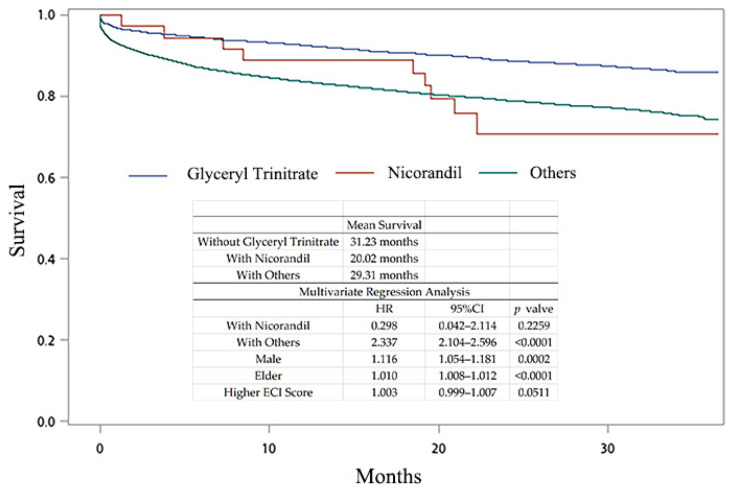
Kaplan–Meier curves of glyceryl trinitrate, nicorandil, and other medications prescribed for hyperlipidemia patients with AMI.

Although our study showed that organic nitrates are beneficial, we hypothesized that the survival benefit of different NO-enhancing agents may not be the same. Nicorandil is a vasodilator used for cardiac diseases, and it also has the ability to enhance NO production [19]. Glyceryl trinitrate is an organic nitrate, and we chose it as a representative to compare with nicorandil. Hyperlipidemia patients with AMI treated without glyceryl trinitrate or nicorandil were classified as the “Others” group. The demographic data of these three groups are shown in Table 4. The patients receiving glyceryl trinitrate were younger and had a lower ECI score. Figure 7 shows the patients’ survival curves (31.23 months with glyceryl trinitrate, 20.02 months with nicorandil, and 29.31 months with others). According to multivariate analysis, other treatments and being male and elderly were associated with a worse survival profile. These results indicate that the efficacies of different NO-enhancing agents are not the same, and some are better candidates for treating hyperlipidemia than others.

## 4. Discussion

This nationwide population-based study was based on the NHIRD in Taiwan and systematically analyzed the outcome data on the differences in treatment options for hyperlipidemia patients with acute myocardial infarction from 2016 to 2018. To the best of our knowledge, this is the largest real-world outcome study for hyperlipidemia patients with acute myocardial infarction in Taiwan in recent years. We determined that most of the patients with hyperlipidemia were 61–70 years old (29.95–31.46% from 2016 to 2018) (Table 1), and these results are similar to those of another study [20]. Among these patients with hyperlipidemia, the annual AMI risk was less than 1% (0.42–0.68% from 2016 to 2018). Among hyperlipidemia patients with AMI, males were predominant, and there was a higher prevalence of AMI in patients aged 61–80 years (Table 2). The all-cause mortality in patients treated without organic nitrates was significantly higher than in those receiving organic nitrate treatment in the unadjusted and PSM analyses (Figure 2 and Figure 4, *p* < 0.0001). In all age groups except age <= 40 years, the patients receiving organic nitrates exhibited significant differences in the all-cause mortality rates compared to those not receiving organic nitrates (Figure 3). Treatments that included beta-blockers resulted in better survival benefits compared to the other treatments. Among the five categorized treatments, the all-cause mortality in the treatments without organic nitrates was significantly higher than in the treatments that involved organic nitrates. These results indicate that treatments that include organic nitrates are better options for hyperlipidemia patients with AMI.

According to the AHA/ACC guidelines, treatment with beta-blockers should be initiated early for patients without contraindications and continued unless adverse effects are observed during the early convalescent phase of ST elevation myocardial infarction (STEMI) [21]. Daisaku et al. reported that beta-blocker treatment is still effective in the long-term outcomes of STEMI survivors without LV systolic dysfunction for secondary prevention after AMI [22]. The benefits of ACEI mainly appeared in higher-risk subgroups, but the survival benefit for patients in low-risk subgroups without these features is unclear [23,24]. In our study population, around 50% of the patients had an age below 65 years, and one-third were male (Table 1). Younger (≤65 years) and male patients were more likely to be classified as low-risk AMI survivors and therefore not benefit as greatly from ACEI treatment as high-risk subgroups, resulting in higher all-cause mortality compared to the groups treated with beta-blockers and other medications (Figure 2) [25].

Nitrates were able to reduce the cardiac preload and enhance the perfusion of ischemic myocardial zones via coronary vasodilation and systemic venodilatation. Several studies have also demonstrated that nitrates result in infarct size reduction [26]. Although nitroglycerin tolerance has been reported, we still found that nitroglycerin administration provided significantly better survival benefits compared to non-nitroglycerin administration groups for hyperlipidemia patients with AMI in this study. Unlike in clinical and experimental settings, in which infarct size measurements are reliable, there are no records for nitroglycerin tolerance, clinical biomarkers, or infract size in retrospective database analysis. It would be very difficult to use endpoints better than survival in this study. AMI is a common cause of heart failure, which can develop soon after AMI or after a longer period of time, and it may persist or resolve [27]. Most deaths after an MI are preceded by the development of HF, and the risk of developing HF is highly associated with mortality. An alternative way to study the outcomes of treatments among hyperlipidemia patients with AMI is to focus on heart failure after AMI.

Although the analyses of the NHIRD data have provided several benefits, such data still have limitations due to the nature of the NHIRD design, and the data are not blinded or randomized. First, there are a lack of data pertaining to self-funded medications, laboratory data, and patient information (height, weight, etc.) because of the objective of the NHIRD. The severity of the disease also cannot be measured. Second, this study design was strictly based on the ICD-10-CM and ICD-10-OP system, since coding errors, misclassifications, and differences among hospitals and physicians can affect these results. Thus, the proportion of our subjects may be incorrect. Third, due to differences in healthcare systems, demographics, and lifestyle factors, our results might not be universally applicable to other regions or countries. Finally, the National Health Insurance Administration needs almost a year to systematically list annual data and release them. Hence, we could not include the most recent patients in our study, and we are still waiting for more data for long-term observation.

## 5. Conclusions

In this study, we found that hyperlipidemia patients with AMI were mostly male, with a higher prevalence at the age of 61–70 years (25.64%). The survival benefit of treatment with organic nitrates was significantly higher than that for treatment that did not involve organic nitrates, especially when combined with diuretics. These results indicate that treatments that include organic nitrates are a better treatment option for hyperlipidemia patients with AMI.

## Figures and Tables

**Table 1:** Demographic data of hyperlipidemia patients.

Year	2016	2017	2018
OPD ≥ 2	2,169,346	2,333,994	2,451,335
IPD	96,284	101,918	108,978
Patients with hyperlipidemia (N) *	2,208,962 (9.28%)	2,374,463 (9.94%)	2,495,158 (10.42%)
Gender			
Female	1,081,974 (48.98%)	1,164,910 (49.06%)	1,226,360 (49.15%)
Male	1,102,406 (49.91%)	1,182,746 (49.81%)	1,238,586 (49.64%)
Unknown	24,582 (1.11%)	26,807 (1.13%)	30,212 (1.21%)
Age			
≤40	131,375 (5.95%)	138,925 (5.85%)	144,687 (5.80%)
41–50	282,721 (12.80%)	300,845 (12.67%)	315,106 (12.63%)
51–60	616,257 (27.90%)	645,965 (27.20%)	662,774 (26.56%)
61–70	661,683 (29.95%)	735,553 (30.98%)	785,010 (31.46%)
71–80	353,718 (16.01%)	374,190 (15.76%)	395,136 (15.84%)
≥81	147,492 (6.68%)	162,233 (6.83%)	173,479 (6.95%)
Unknown	15,716 (0.71%)	16,752 (0.71%)	18,966 (0.76%)
Acute myocardial infarction (N)	14,688 (0.66%)	16,034 (0.68%)	10,388 (0.42%)
	(665 per 100,000)	(675 per 100,000)	(416 per 100,000)

*: The annual patients with hyperlipidemia divided by the total population of index year.

**Table 2:** Demographic data of hyperlipidemia patients with AMI and their medication usage.

	Without Organic Nitrates					With Organic Nitrates				
	ACEIs/ ARB (17)	Beta-Blockers (552)	Beta-Blockers + Diuretics (223)	Diuretics (496)	Others (28,456)	ACEIs/ ARB (72)	Beta- Blockers (4312)	Beta- Blockers + Diuretics (2360)	Diuretics (1263)	Others (3359)
Gender										
Male	7 (41.18%)	408 (73.91%)	136 (60.99%)	286 (57.66%)	19,909 (69.96%)	57 (79.17%)	3553 (82.40%)	1656 (70.17%)	848 (67.14%)	2531 (75.35%)
Female/Unknown	10 (58.82%)	144 (26.09%)	87 (39.01%)	210 (42.34%)	8442 (29.67%)	15 (20.83%)	759 (17.6%)	704 (29.83%)	415 (32.86%)	828 (24.65%)
Age										
≤40	0 (0.00%)	18 (3.26%)	54 (24.22%)	5 (1.01%)	931 (3.27%)	3 (4.17%)	247 (5.73%)	47 (1.99%)	15 (1.19%)	147 (3.48%)
41–60	3 (17.65%)	234 (42.39%)		78 (15.73%)	8865 (31.15%)	24 (33.33%)	2011 (46.64%)	686 (29.07%)	266 (21.06%	1272 (37.87%)
61–80	10 (58.82%)	239 (43.30%)	106 (47.53%)	251 (50.60%)	13,698 (48.14%)	34 (47.22%)	1761 (40.84%)	1190 (50.42%)	653 (51.70%)	1565 (46.59%)
≥81	4 (25.53%)	61 (11.05%)	63 (28.25%)	162 (32.66%)	4962 (17.44%)	11 (15.28%)	293 (6.79%)	437 (18.52%)	329 (26.05%)	375 (11.16%)
Mean	72.41	62.96	70.97	73.18	66.19	63.77 *	60.09 ***	67.20 ***	70.80 ***	63.31 ***
Std	9.82	13.08	13.20	12.76	13.73	13.08	12.61	13.14	12.74	13.24
Comorbidity										
Congestive heart failure	4 (23.53%)	112 (20.29%)	127 (53.95%)	326 (65.73%)	10,317 (36.26%)	12 (16.67%)	804 (18.65%)	1355 (57.42%)	877 (69.44%)	843 (25.10%) ***
Cardiac arrhythmias	5 (29.41%)	120 (21.74%)	64 (28.70%)	156 (31.45%)	6078 (21.36%)	14 (19.44%)	679 (15.75%) ***	567 (24.03%)	344 (27.24%)	681 (20.27%)
Valvular disease		30 (5.43%)	28 (12.56%)	65 (13.10%)	2362 (8.30%)	3 (4.17%)	201 (4.66%)	224 (9.49%)	164 (12.98%)	267 (7.95%)
Hypertension, uncomplicated	15 (88.24%)	373 (67.57%)	159 (71.30%)	329 (66.33%)	18,554 (65.20%)	66 (91.67%)	2766 (64.15%)	1566 (66.36%)	853 (67.54%)	2173 (64.69%)
Hypertension, complicated	10 (58.82%)	194 (35.14%)	106 (47.53%)	261 (52.62%)	12,927 (45.43%)	33 (45.83%)	1447 (33.56%)	1147 (48.60%	676 (53.52%)	1287 (38.31%) ***
Diabetes, uncomplicated	3 (17.65%)	72 (13.04%)	49 (21.97%)	132 (26.61%)	5367 (18.86%)	8 (11.11%)	515 (11.94%)	497 (21.06%)	333 (26.37%)	600 (17.86%)
Diabetes, complicate	10 (58.82%)	210 (38.04%)	126 (56.50%)	288 (58.06%)	13,615 (47.85%)	44 (61.11%)	1691 (39.22%)	1286 (54.49%) *	707 (55.98%)	1416 (42.16%) ***
Renal failure	0 (0.00%)	7 (1.27%)	3 (1.35%)	11 (2.22%)	380 (1.34%)	≤3 (≤4.2%)	49 (1.14%)	35 (1.48%)	25 (1.98%)	42 (1.25%)
ECI score										
Mean	12.53	10.75	15.72	17.36	13.02	10.56	10.07 **	14.76 *	16.63 *	11.34 ***
Std	6.52	6.15	6.71	7.32	7.12	5.44	5.73	6.91	6.83	6.65

The differences in age, comorbidity, and ECI score among five categories with or without organic nitrates were tested. * *p* < 0.05, ** *p* < 0.01 and *** *p* < 0.001.

**Table 3:** Demographic data of hyperlipidemia patients with AMI after PSM (gender, age, and ECI score).

	Without Organic Nitrates	With Organic Nitrates
Gender		
Male	8357 (77.01%)	8357 (77.01%)
Female/Unknown	2495 (22.99%)	2495 (22.99%)
Age		
≤40	383 (3.53%)	383 (3.53%)
41–60	4056 (37.38%)	4056 (37.38%)
61–80	5069 (46.71%)	5069 (46.71%)
≥81	1344 (12.38%)	1344 (12.38%)
Mean	63.90	63.90
Std	13.09	13.09
Comorbidity		
Congestive heart failure	3524 (32.47%)	3641 (33.55%)
Cardiac arrhythmias	2040 (18.80%)	2135 (19.67%)
Valvular disease	706 (6.51%)	765 (7.05%)
Peripheral vascular disorders	350 (3.23%)	346 (3.19%)
Hypertension, uncomplicated	7007 (64.57%)	7125 (65.66%)
Hypertension, complicated	4587 (42.27%)	4359 (40.17%) **
ECI Score		
Mean	11.91	11.91
Std	6.34	6.34
Median	11	11

The differences in age, comorbidity, and ECI score among patients with or without organic nitrates were tested. ** *p* < 0.01.

**Table 4:** Demographic data of glyceryl trinitrate, nicorandil, and other medications prescribed for hyperlipidemia patients with AMI.

	Glyceryl Trinitrate	Nicorandil	Others
Patient Number	3954 (9.62%)	36 (0.09%)	37,120 (90.29%)
Gender			
Male	3130 (79.16%)	29 (80.56%)	26,232 (70.67%)
Female	824 (20.84%)	7 (19.44%)	10,888 (29.33%)
Age			
≤40	208 (5.26%)	0 (0.00%)	1206 (3.25%)
41–60	1677 (42.41%)	16 (44.44%)	11,799 (31.79%)
61–80	1665 (41.11%)	13 (36.11%)	17,829 (48.03%)
≥81	404 (10.22%)	7 (19.44%)	6286 (16.93)
Mean	61.90	65.61	65.97 ***
Std	13.38	13.79	13.67
Comorbidity			
Congestive heart failure	1186 (29.99%)	19 (52.78%) **	13,572 (36.56%) ***
Cardiac arrhythmias	700 (17.70%)	16 (44.44%)	7994 (21.54%) ***
Valvular disease	286 (7.23%)		3057 (8.24%) *
Pulmonary circulation disorders	23 (0.58%)		398 (1.07%) **
Peripheral vascular disorders	149 (3.77%)		1513 (4.08%)
Hypertension, uncomplicated	2580 (65.25%)	28 (77.78%)	24,244 (65.31%)
Hypertension, complicated	1458 (36.87%)	17 (47.22%)	16,613 (44.75%) ***
Other neurological disorders	32 (0.81%)		562 (1.51%) ***
Chronic pulmonary disease	3884 (98.23%)	36 (100.00%)	36,430 (98.14%)
Diabetes, uncomplicated	623 (15.76%)	5 (13.89)	6948 (18.72%) ***
Diabetes, complicate	1664 (42.08%)	18 (50.00%)	17,711 (47.71%) ***
Renal failure	46 (1.16%)	4 (11.11%) ***	503 (1.36%)
ECI Score			
Mean	11.35	14.42 **	12.97 ***
Std	6.58	7.40	7.09

The difference in age, comorbidity, and ECI score of patients prescribed glyceryl trinitrate compared with patients prescribed nicorandil or other treatments. * *p* < 0.05, ** *p* < 0.01 and *** *p* < 0.001.

## Data Availability

The data underlying this study belong to the National Health Insurance Research Database (NHIRD) of Taiwan and cannot be made publicly available due to legal restrictions. However, the data are available through a formal application to the Health and Welfare Data Science Centre at the Ministry of Health and Welfare, Taiwan (https://dep.mohw.gov.tw/DOS/np-2500-113.html, accessed on 1 November 2023), and require a signed affirmation regarding data confidentiality. The authors have no special privilege of access to the database.
